# Renal cell carcinoma in a transplanted kidney: a retrospective evaluation

**DOI:** 10.1186/s12882-024-03673-0

**Published:** 2024-07-29

**Authors:** Ruslan N. Trushkin, Teymur K. Isaev, Pavel E. Medvedev, Nikolai E. Shcheglov, Valerii V. Variasin, Mariana A. Lysenko, Ilya V. Dmitriev, Aslan G. Balkarov, Laura A. Kesaeva

**Affiliations:** 1 Department of Urology, Moscow City Clinical Hospital № 52 of Moscow Healthcare Department, Moscow, Russian Federation; 2 Department of Kidney and Pancreas transplantation, Sklifosovsky Research Institute for Emergency Medicine of Moscow Healthcare Department, 3 Bol’shaya Sukharevskaya Sq., Moscow, 129090 Russian Federation; 3 Chair of Transplantology and Artificial Organs, Department of Continuous Medical Education, Pirogov Russian National Research Medical University, Ministry of Health of the Russian Federation, Moscow, Russian Federation; 4 Limited Liability Company “GenoTechnology”, Moscow, Russian Federation

**Keywords:** Kidney graft neoplasm, Renal cell carcinoma of the kidney graft, Laparoscopic resection of kidney graft tumor, Genetic study of renal cell carcinoma in kidney transplant

## Abstract

**Introduction:**

Kidney transplantation is the optimal treatment modality for patients with end-stage chronic kidney disease. The long-term mortality of kidney recipients is 48–82% lower than that of patients on the waiting list. However, the risk of developing malignancies in these patients is twice as high as in the healthy population. Specifically, the incidence of renal cell carcinoma (RCC) in transplant recipients is 10–30 times higher than in non-transplanted patients. The reason for the increased risk is poorly understood, but is most likely related to continuous immunosuppressive therapy. The problem of kidney graft neoplasia has not been adequately addressed in the medical literature.

**Objective:**

To determine the incidence of renal cell carcinoma in transplanted kidneys, enhance the efficacy of its treatment, and study the etiology of RCC development.

**Materials and methods:**

A retrospective analysis of RCC incidence in kidney grafts was conducted in 3,270 patients who underwent kidney transplantation between 2013 and 2023. We evaluated the effectiveness of surgical interventions for these complications. Patients with histologically confirmed RCC of the transplanted kidney underwent genetic study to determine the etiology of the neoplasm.

**Results:**

The incidence of RCC in transplanted kidneys was found to be 0.95% (*n* = 31), 28 patients underwent laparoscopic resection of the renal transplant tumor, 2 patients were treated with radiofrequency ablation of the tumor. Transplantectomy was performed in 1 patient.

**Conclusion:**

Laparoscopic resection is an effective and safe method for the treatment of RCC in kidney transplants. Transplanted kidney cancer originates from the donor tissue. The clear cell variant of transplanted kidney cancer is a genetically determined disease.

## Introduction

According to the Russian Registry of Renal Replacement Therapy (RRT) for End-Stage Chronic Kidney Disease (ESKD) of the Russian Dialysis Society, approximately 60,000 patients in Russia are currently receiving some form of RRT, 17% of whom are kidney transplant recipients. Annually, more than a thousand patients in Russia become kidney transplant recipients, and there is a clear trend toward increasing the number of kidney transplants each year [1]. Despite advances in comprehensive post-transplant care, long-term survival of kidney grafts and patients remains low in some cases. For instance, about 40–42% of transplanted kidneys fail within ten years after the transplantation [3].

Despite the increasing number of publications on malignant lesions of the transplanted kidney over the past decades, there are few studies on renal cell carcinoma (RCC) of the kidney graft due to the rare occurrence of this disease. In one of the largest meta-analyses dedicated to renal transplant malignancies by Griffith, J.J. et al., the potential incidence of RCC in transplant recipients was reported to be between 0.19 and 0.5%, which is 10–30 times higher than the frequency observed in the non-transplanted population. In the modern literature on renal transplant malignancies, a total of approximately 200 patient outcomes have been described. Treatment has been limited to ablative methods, with isolated cases of minimally invasive resection of RCC in transplanted kidneys reported [5]. Therefore, due to the insufficient data on the surgical treatment of RCC in transplanted kidneys and its outcomes, and the lack of analysis on the etiology of the neoplasm[10], the present study was conducted to fill this gap.

## Materials and methods

### Kidney transplant recipients

3,270 patients at various times after kidney transplantation were monitored by specialists of the Moscow City Nephrology Center from 2013 to 2023. There were no macroscopic signs of RCC of the kidney graft at organ recovery, during work up and before transplantation. The frequency of the imaging of the transplanted kidney was determined according to local post-transplant monitoring protocols (for an uncomplicated postoperative period - at least once a month during the first year after transplantation, then every 2–3 months a year). RCC of the transplanted kidney was identified and histologically confirmed in 31 recipients. This group included 16 male patients (51.6%) and 15 female patients (48.4%), with ages ranging from 26 to 69 years. The median age was 50.5 years, with an interquartile range of 43.3 to 60.5 years. Primary kidney transplantation had been performed in all patients: 30 patients (94.1%) received their transplant from deceased donors, and one patient received a kidney from a living related donor.

### Surgical treatment

We applied a standardized technique for performing laparoscopic resection of tumor-affected transplanted kidneys: laparoscopic isolation of the renal graft was performed, followed by identification of the neoplasm in the transplanted kidney. Temporary ischemia of the graft was induced by clamping the external iliac artery with an endoscopic vascular clamp above the implantation zone of the renal hilum. The tumor was then removed with high-energy instruments, and the defect in the renal parenchyma was closed with knotted intracorporeal barbed sutures. Intraoperative ultrasound guidance was used in cases where visualization of the tumor location was challenging.

Radiofrequency ablation of malignant neoplasms in transplanted kidneys was performed with the Metatom-2 universal system, following the standard protocol used for ablation of native kidney lesions. Sessions of monopolar radiofrequency thermal ablation were performed with a power range of 30 to 40 watts, achieving an average temperature of 72 °C at the tumor lesion site.

### Immunosuppressive therapy

Patients in the study group received standard triple-component baseline immunosuppressive therapy (IST), which in majority of the recipients (*n* = 30, 96.8%) included calcineurin inhibitors, mycophenolic acid agents, and glucocorticosteroids. Tacrolimus was used as the calcineurin inhibitor in 22 (71%) patients, while cyclosporine was used in 8 (25.8%). In one patient, IST included the mammalian target of rapamycin (mTOR) inhibitors instead of mycophenolic acid agents. Upon detection of RCC in the kidney graft, recipients (*n* = 21, 67.7%) were converted from mycophenolic acid to everolimus.

Since the study group included patients with kidney transplantations performed in different centers and from 4 to 16 years prior to this study, it was not feasible to obtain information on the induction immunosuppressive therapy administered at the time of transplantation.

### Histological examination technique

Morphologic examination of the resected segment of the tumor-bearing kidney graft was performed according to standard protocol in all patients. Tissue fixation was performed in a 10% solution of neutral buffered formalin, followed by rinsing with water and dehydration through a graded series of alcohols of increasing concentration, then embedded in paraffin. Sections of 5 μm thickness were cut from the paraffin blocks and stained with hematoxylin and eosin. Histological examination was performed with a light microscope (Nikon Y-TV55, Japan) in both transmitted and polarized light.

### Methods for genetic studies

A genetic analysis of the tumor and graft tissue in the transplanted kidney was conducted. DNA was extracted from paraffin-embedded blocks. Chimerism assessment was performed using polymerase chain reaction (PCR) with electrophoretic detection, based on the analysis of chromosomal DNA. This method identifies and compares specific genetic markers known as short tandem repeats (STRs) in various loci within the tissue samples of the transplanted kidney and the tumor. Each person inherits two STR sequences within a single locus, and STRs occur at different loci with different frequencies in the population. Thus, analyzing a greater number of loci allows for the identification of more informative alleles for chimerism analysis. The number of loci analyzed is crucial in chimerism testing. We tested 7 STR loci: FGA (FIBRA), D8S639, THO1 (TC11), HUNvWFA31, D19S246, D21S11, D18S51. Additionally, the VHL gene in the transplanted kidney tumor was analyzed. The material was obtained from paraffin blocks following histological evaluation. DNA sequencing was performed using the Sanger method, particularly focusing on the most common mutations in exons 1, 2, and 3 of the VHL gene.

A genetic study was possible in patients with sufficient volume of tissue in paraffin blocks for DNA isolation.

### Statistical processing

Statistical data analysis was conducted using the Statistica for Windows v.12.0 software package by StatSoft Inc. (USA). Data were presented as median (Me) and interquartile ranges (Q1-Q3). The differences were considered statistically significant at *p* < 0.05.

## Results

The follow-up period of the patients was as follows 3.6 [2.1–5.3] years.

Among all patients who underwent kidney transplant and were monitored at the Moscow City Nephrology Center, the proportion of adult men was 57.52% (1881 people), while the female proportion was 42.48% (1389 people). The age of the patients ranged from 18 to 84 years, with an average of 41.3 ± 12.5 years. It is also worth noting that among patients with verified cancer of a transplanted kidney, the proportion of men was 51.6% (16 people), and women − 48.4% (15 people). The age distribution of patients with RCC allograft was predominantly within the range of 26 to 69 years, with an average age of 51.8 ± 8.5 years.

### Incidence of RCC in a transplanted kidney

The overall incidence of renal cell carcinoma (RCC) in kidney transplants was 0.95%. All RCC were detected in the long-term period after kidney transplantation, the time from transplantation to RCC development ranged from 2 to 25 years, with an average of 8.4 years. The size of the RCC was 24 [17–30] mm. Pathomorphological examinations revealed clear-cell RCC in 20 patients (64.5%) and papillary RCC in 11 patients (34.5%). The average time to the development of clear-cell RCC was 9.9 years, compared to 5.5 years for papillary RCC.

### Surgical treatment of RCC in transplanted kidney

According to the RCC treatment protocols used in our center, chemotherapy and/or immunotherapy before surgical or ablative treatment were not performed in any patient of the study group. The surgical treatment was performed in 29 patients (94%): 28 patients underwent laparoscopic organ-preserving resection of the neoplasm-bearing kidney graft, histologically it was confirmed that the resection was performed within healthy tissues; one patient underwent laparoscopic transplantectomy due to extensive tumor involvement. The median duration of surgical intervention in the patient group with intraoperative minimally invasive ultrasound navigation was 135 min (117.5–144 min), compared to 123 min (112.5–144.5 min) in the group without navigation. The median ischemia time was significantly lower in the ultrasound guidance group at 24 min (20–25 min) versus 29 min (24–32 min) in the group without additional imaging (*p* < 0.05). Intraoperative blood loss ranged from 50 to 500 ml, and averaged approximately 162 ml. In case of opening of the collecting system of the kidney, the defect was also sutured with intracorporeal interrupted sutures. In one case, given the large size of the tumor, the calyx was cut off from the fornix, followed by the formation of a fornical-calyceal anastomosis. After laparoscopic resection of the transplanted kidney with the tumor, all patients experienced maintained function of the kidney transplant, despite a transient increase in serum nitrogenous base levels and a reduction in urine output. Specifically, the average serum creatinine level prior to surgical treatment and 12–18 h post-laparoscopic resection were 187.1 µmol/L and 232.4 µmol/L, respectively (*p* < 0.05). A decrease in average creatinine levels to nearly preoperative values was observed on postoperative day 6 and 7, reaching 192.5 µmol/L. No recurrence of RCC in the area of resection was observed in any of the patients during the follow-up period.

### Genetic insights

**Fig. 1:**
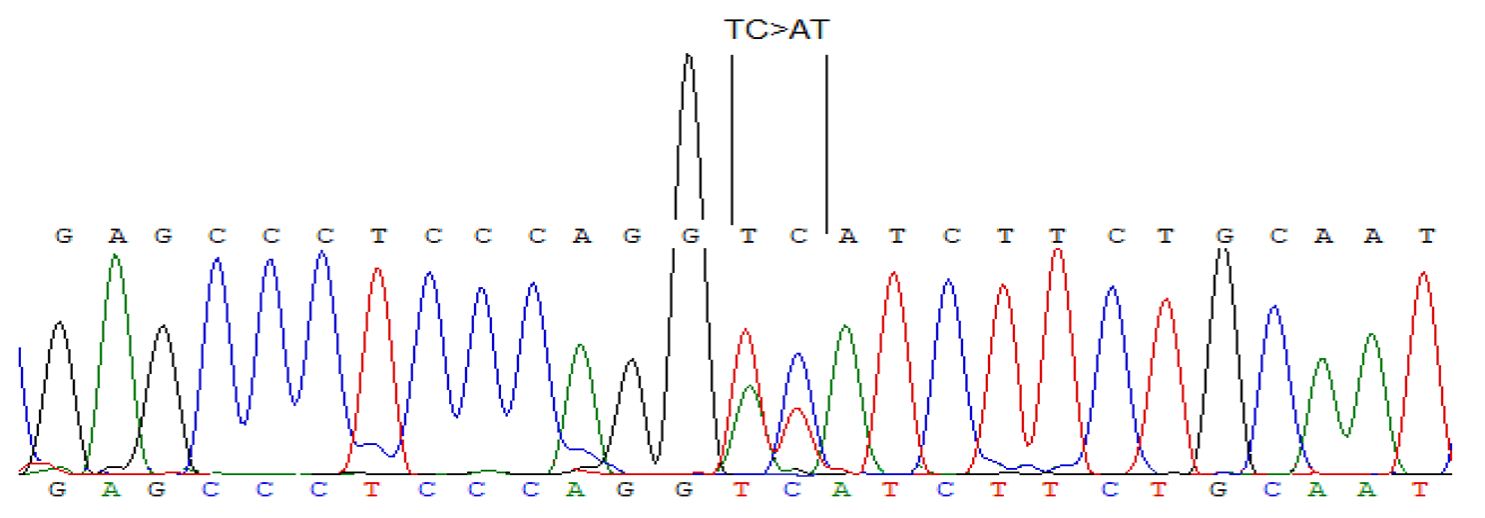
Mutation in the 1st exon of the Von Hippel–Lindau gene, substitution c.221_222 TC > AT

The extraction of DNA from paraffin-embedded blocks of tumor and transplanted kidney tissue, obtained at post-histological examination, was successful in only 11 out of 31 patients. A 100% match was observed between the DNA markers of the transplanted kidney tumor and the DNA from the unaffected transplanted kidney tissue. A study of the VHL gene in the context of clear-cell RCC in transplanted kidneys was possible in 7 tissue samples. Mutations in the exons of VHL gene were identified in 5 of these samples (71.4%). Specifically, a c.221_222 TC > AT substitution in exon 1 of the VHL gene was detected, leading to a p.Val74Asp amino acid substitution (Fig. 1).

**Fig. 2:**
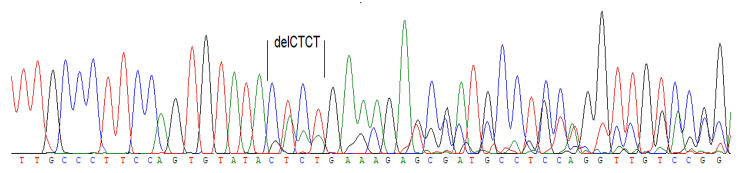
Mutation in the 3rd exon of the Von Hippel–Lindau gene, deletion c.470_473delCTCT

Deletion of c.470_473delCTCT (p.Thr157Arg*fs12) was found in exon 3 of the VHL gene, resulting in a frameshift mutation. This alteration leads to the substitution of the C-terminal peptide with a different amino acid sequence and the premature introduction of a stop codon. (Fig. 2).

The identification of VHL gene mutations in the tumor DNA, corresponding to the donor genotype, established that the cancer in the transplanted kidney originated from an inherent genetic predisposition of the transplant’s renal parenchyma, i.e. the donor tissue, to the development of RCC. Such predisposition was likely exacerbated by the continuous administration of immunosuppressive therapy. In patients with a VHL gene mutations, the timing of RCC onset was earlier compared to patients without VHL gene mutations (4[3–19] years vs. 7[5–14] years), however, no statistical significance was noted (*p* = 0.76).

The average time to RCC development in patients receiving tacrolimus as IST primary component was statistically shorter compared to those receiving cyclosporine, with median times of 5 years (3.8–6.3 years) and 14 years (8.5–16 years), respectively (*p* < 0.05).

## Discussion

Transplantation remains the most effective treatment for patients with stage 5 CKD, as it provides the best possible life expectancy and quality of life for this patient group. According to the registry data, there were 9984 patients with a functioning kidney transplant in the Russian Federation in 2020 [2]. Advances in post-transplant treatment protocols contribute to an increase in the number of patients with long-term functioning kidney transplants.

Oncological complications, including malignant neoplasms of the transplanted kidney, are among the most frequent complications during the extended post-transplant period. A meta-analysis by Griffith, J.J. et al. reported the incidence of RCC in transplant recipients to be between 0.19% and 0.5%. In our cohort, the incidence of RCC was 0.95%. Malignant tumors of the transplanted kidney were represented mainly by clear-cell RCC (*n* = 20, 64.5%) and papillary RCC (*n* = 11, 34.5%), with their development averaging 9.9 years and 5.5 years, respectively.

While some studies have focused on ablative treatment methods, with isolated cases of minimally invasive RCC resection [5], we believe the coverage of surgical intervention for RCC and its outcomes is insufficient. Our findings suggest that laparoscopic resection of the tumor-bearing transplanted kidney is an effective and relatively safe method for organ-preserving treatment of malignant neoplasms.

Our analysis showed that the use of tacrolimus was associated with a quicker development of RCC compared to cyclosporine (5 years vs. 13 years, respectively; *p* < 0.05). We also found a 100% concordance between the DNA of the transplanted kidney tumor and the DNA of the unaffected transplanted kidney tissue based on the analyzed markers. Furthermore, a mutation in the VHL gene was present in over 71% of clear-cell RCC cases. The findings suggest that comprehensive IST may stimulate endothelial growth factors [8], contributing to the oncogenesis in genetically modified transplanted kidney tissues. Future genetic examination of donor genomes for VHL and MET mutations could be crucial for determining genetic predispositions to clear cell and papillary RCC in transplanted kidneys as well as for selecting effective IST.

### Limitations

The retrospective nature of the study inherently limits the ability to establish causality. Such designs are more prone to biases and confounding factors that cannot be controlled as effectively as in prospective studies. Genetic analysis was conducted on a limited subset of patients (11 out of 31), which may not provide a comprehensive view of the genetic landscape of RCC in transplanted kidneys. This small sample size limits the generalizability of the genetic findings. Given the study’s retrospective design and the specific patient population from a single geographic location, the results may not be generalizable to all kidney transplant recipients. Selection bias could influence the incidence rate of RCC and the observed outcomes of surgical interventions. The absence of a control group of kidney transplant recipients without RCC limits the ability to compare outcomes directly and may affect the interpretation of the effectiveness of the surgical treatments and the impact of immunosuppressive therapy. The study may not have controlled for all potential confounding factors, such as variations in immunosuppressive therapy regimens, patient comorbidities, and lifestyle factors that could influence the development of RCC and the outcomes of treatment. The genetic findings, particularly regarding the VHL gene mutations, are based on a small number of cases. This limitation restricts the ability to generalize these results to all cases of RCC in transplanted kidneys. As stated, we were unable to obtain information on the induction immunosuppressive therapy administered at the time of transplantation for all patients, which could be relevant for understanding the development of RCC. The study primarily focuses on the immediate and short-term outcomes of surgical interventions for RCC in transplanted kidneys. Long-term outcomes, including survival, graft function, and recurrence of RCC, are not thoroughly addressed.

## Conclusions

1. In our study, the incidence of renal cell carcinoma in kidney transplant recipients reached 0.97%, which is higher that that reported in other similar studies.
2. Renal cell carcinoma of a transplanted kidney originated from donor tissue. In clear cell cancer of a transplanted kidney, a mutation of the Von Hippel–Lindau gene was detected in 71.42% of cases, which reflects the presence of genetic determination of the malignant process.
3. The novel technique for laparoscopic resection of a kidney graft with a tumor developed within the study is safe and effective method of treatment of allograft RCC.

## Data Availability

The datasets used and/or analysed during the current study available from the corresponding author on reasonable request.
